# Alterations in expression of endometrial milk fat globule-EGF factor 8 (MFG-E8) and leukemia inhibitory factor (LIF) in patients with infertility and endometriosis

**DOI:** 10.5935/1518-0557.20170056

**Published:** 2017

**Authors:** Carla Regina Schmitz, Sergio Oehninger, Vanessa Krebs Genro, Neelima Chandra, Frank Lattanzio, Liang Yu, João Sabino Cunha-Filho

**Affiliations:** 1 Graduate Program in Internal Medicine of the Universidade Federal do Rio Grande do Sul, Porto Alegre, RS, Brazil; 2 Department of Obstetrics and Gynecology, Eastern Virginia Medical School, The Jones Institute for Reproductive Medicine, Norfolk VA, USA; 3 Department of Obstetrics and Gynecology, Universidade Federal do Rio Grande do Sul, Porto Alegre, RS, Brazil; 4 Department of Physiological Sciences, Eastern Virginia Medical School, Norfolk VA, USA

**Keywords:** MFG-E8, integrin αvβ3, LIF, human endometrium, endometriosis

## Abstract

**Objective:**

The aim of this study was to compare the endometrial expression of milk fat globule-EGF factor 8 (MFG-E8), its receptor integrin αvβ3, and leukemia inhibitory factor (LIF) in patients with endometriosis and infertility and in healthy fertile patients during the window of implantation.

**Methods:**

Five patients with peritoneal endometriosis and infertility (case group) and four healthy fertile patients (control group) were recruited. All patients were either diagnosed with or ruled out for endometriosis by laparoscopic surgery; the case group underwent surgery for infertility investigation and the control group for tubal ligation. Endometrial biopsies were performed in all patients during the window of implantation (LH+8 to LH+10), and then the samples were analyzed by immunochemistry for MFG-E8, integrin αvβ3, and LIF.

**Results:**

In patients with endometriosis and infertility, expression of MFG-E8 was significantly increased in the glandular epithelium when compared to healthy fertile patients (*p*<0.001). Moreover, LIF expression was lower in patients with endometriosis and infertility (*p*<0.05). Nevertheless, we found no difference in integrin αvβ3 expression between the groups (*p*=0.084).

**Conclusion:**

This study showed for the first time that MFG-E8 expression is impaired in the endometrium of patients with endometriosis and infertility during the window of implantation. Moreover, LIF is also diminished in the endometrium of these patients as shown before.

## INTRODUCTION

Endometriosis is one of the most common gynecologic diseases, and its clinical manifestations include dyspareunia, dysmenorrhea, chronic pelvic pain, and infertility (Howard, 2009; ASRM, 2006). It is present in approximately 10% of women in fertile age (Härkki *et al*., 2010), but among infertile patients, endometriosis prevalence can be as high as 25-50% (Dong *et al*., 2013). Although it is known for decades that endometriosis is associated with infertility, the pathogenesis of this association is still not completely understood (Härkki et al., 2010). Nevertheless, several studies have shown that endometriosis is associated with abnormal folliculogenesis, luteal insufficiency (Cunha-Filho *et al*., 2003; 2001), and abnormal embryo fertilization and implantation (Fadhlaoui *et al*., 2014), which contribute to infertility. Regarding lower implantation rates, it has been shown that some endometrial receptivity markers may be impaired in the endometrium of these patients (Lu *et al*., 2013; Dimitriadis *et al*., 2006; Dong et al., 2013).

Leukemia inhibitory factor (LIF) and integrin αvβ3 are well-known endometrial receptivity markers. LIF is a polyfunctional pleiotropic cytokine, which belongs to the IL-6 family (Tawfeek *et al*., 2012). Stewart *et al*. (1992) have shown for the first time that mice lacking a functional LIF gene fail to implant blastocysts in their endometrium. Since then, many authors have demonstrated that proper LIF expression by the endometrium is also important for female fertility (Lalitkumar *et al*., 2013; Mikolajczyk *et al*., 2007). LIF is expressed mainly in the glandular and luminal epithelium of the endometrium during the window of implantation (Vogiagis *et al*., 1996). Impaired LIF expression has been shown in patients with unexplained infertility, uterine anatomical abnormalities, and endometriosis (Mikolajczyk et al., 2007; Hasegawa *et al*., 2012; Dimitriadis *et al*., 2006).

Integrin αvβ3 is a transmembrane glycoprotein that has been extensively studied in the human endometrium. It can be detected in the epithelial layer of mice and the human endometrium during the window of implantation (Apparao *et al*., 2001; Liu *et al*., 2013; Franchi *et al*., 2011). Several studies have demonstrated that decreased expression of this protein can impair embryo implantation in vitro (Zhang *et al*., 2011; Kaneko *et al*., 2011; Kang *et al*., 2014; Schmitz *et al*., 2014). Moreover, patients with hydrosalpinx, unexplained infertility and recurrent pregnancy loss have presented impaired integrin αvβ3 expression (Daftary *et al*., 2007; Tei *et al*., 2003; Germeyer *et al*., 2014). Nevertheless, the relationship between this integrin and endometriosis has been controversial in the literature (Lessey *et al*., 1994; Ordi *et al*., 2003; Casals *et al*., 2012).

Milk fat globule epidermal growth factor 8 (MFG-E8) is a novel protein recently associated to the implantation process (Mirkin *et al*., 2005). Franchi *et al.* (2011) demonstrated for the first time that MFG-E8 is expressed in the human endometrial epithelium and that it is up-regulated during the window of implantation. Besides, MFG-E8 histological sequence in epithelial cell location suggests luminal secretion of the protein (Franchi et al., 2011). Moreover, we have demonstrated that blocking this protein in an in vitro trophoblast/endometrial epithelium model can impair the implantation process (Schmitz *et al*., 2014). Nevertheless, this protein has never been studied in the endometrium of infertile patients.

Ultimately, what constitutes adequate expression of endometrial receptivity markers integrin αvβ3 and LIF is still controversial in endometriosis patients, while MFG-E8 has not yet been studied in these patients. Considering that endometriosis patients may have an impaired implantation process, this study aimed to compare the endometrial expression of MFG-E8, integrin αvβ3, and LIF between patients with infertility and endometriosis and healthy fertile patients (controls) during the window of implantation.

## MATERIALS AND METHODS

### Design

This prospective case-control study was carried out in the Department of Gynecology of the Hospital de Clínicas de Porto Alegre and in the Jones Institute for Reproductive Medicine. The STROBE guideline was used (von Elm *et al*., 2007).

### Subjects

Five patients with peritoneal endometriosis and infertility (case group) and four healthy fertile patients (control group) were recruited between January 2014 and November 2014 to take part in the study. Diagnosis of infertility was considered when the couple had not conceived after 12 months of contraceptive-free intercourse (ASRM, 2008). The case group included consecutive patients diagnosed with peritoneal endometriosis during laparoscopic surgery meeting the enrollment criteria described below. The degree of endometriosis was categorized based on to the revised classification of endometriosis of the American Society for Reproductive Medicine (ASRM, 1997). Patients submitted to elective laparoscopic tubal ligation were invited to join the control group. These individuals were ruled out for endometriosis (by laparoscopy), had a history of normal fertility, and were non-smokers.

The individuals in the case and control groups had to meet the following enrollment criteria: (i) age between 25 and 38 years (ii) regular menstrual cycles, (iii) presence of both ovaries, (iv) no endocrine disorder and (v) no family history of genetic disease. The case group also had normal sperm analysis. Patients with abnormal ovarian reserve (antral follicle count under 10), obesity (BMI ≥30), history of miscarriage, and smokers were excluded.

The local ethics committee approved this study and a written informed consent was provided to all subjects prior to sample collection (IRB equivalent).

### Endometrial samples

Endometrial biopsies were performed during the natural cycle during the putative window of implantation (LH+8 to LH+10). LH + 1 was considered the day of ovulation (Kao *et al*., 2003). Ovulation was detected by serial ultrasound exams, and it was defined as the 24-h period that separated the identification of a mature, pre-ovulatory follicle on one scan and either of the following on the next scan: (i) follicle rupture; (ii) presence of an early corpus luteum; (iii) presence of free fluid in the cul-de-sac (Ecochard *et al*., 2013).

The biopsies were performed with a Pipelle^®^ catheter (CCD, Paris, France). Each endometrial biopsy specimen was fixed in formalin and embedded in paraffin in preparation for histological examination and detection of MFG-E8, integrin αvβ3, and LIF by immunostaining.

### Immunohistochemistry

Paraffin-embedded tissue blocks of the endometrial biopsy specimens were cut into 5-µm sections. Immunohistochemistry was performed as previously described (Chandra *et al*., 2013). Briefly, the slides were deparaffinized, dehydrated, and rehydrated followed by immersion in retrieval solution 1:10 (Dako). Endogenous peroxidases were quenched with 3% hydrogen peroxide for 10 min and non-specific binding sites were blocked with 1.5% normal goat or horse serum (Vector Laboratories) for 30 min at room temperature. The sections were then covered by appropriate dilutions of primary antibody, MFG-E8 (Abcam) 1:100, integrin αvβ3 (Santa Cruz Biotechnologies) 1:10 or LIF (Sigma) 1:750, and placed in a refrigerator overnight. After primary antibody incubation, the sections were washed with PBS and incubated with anti-mouse or anti-rabbit secondary antibody (Vector Lab) at a dilution of 1:200 for 30 min at room temperature. After incubation with secondary antibodies, the tissues were incubated with ABC reagent (Vector Laboratories) for 30 min, followed by PBS wash. The antigens were localized by incubation with AEC chromogen-substrate (skyTek Labs) and finally mounted with Accergyl mounting media (Accurate Chemicals) with a cover slip. Negative controls included sections treated with a similar dilution of a non-immune IgG1 (isotype control, eBioscience, San Diego, CA, USA). Representative images were photographed with an Olympus BX50 microscope using an Olympus DP70 Q-color 3 camera (Franchi *et al*., 2011).

The assessment of staining intensity and distribution for integrin and LIF was made using the semi-quantitative histologic score (HSCORE) system. The HSCORE was calculated using the following equation: HSCORE: Σ Pi (i + 1), where i represents the intensity of staining on a scale from 1 to 3 (1 for weak, 2 for moderate, and 3 for strong staining) and Pi the percentage of stained endometrial stromal and epithelial cells for each intensity, varying from 0-100%, as previously described (Lessey *et al*., 2006).

The assessment for staining intensity and distribution for MFG-E8 was made using computerized image analysis using a modification of Fuhrich *et al*. (2013), with the aid of Metamorph^TM^ (Molecular Devices) instead of software program Image J. The original method was found to be highly correlated with HSCORE values obtained by expert evaluators. Color images collected using an Olympus 20x objective were automatically thresholded rather than manually circumscribed, and the thresholded areas from three different 20x fields were averaged and subtracted from total white (255) values on an eight byte scale, as published earlier (Fuhrich et al., 2013). A size filter setting was used to exclude stray pixels so that only cell structures were analyzed.

### Statistical analysis

The statistical analysis was carried out using software package SPSS 18.0. The measure of central tendency used was the mean and the measure of variability was the standard deviation (Lambalk *et al*., 2004). Categorical variables in the two groups were compared using the 2-sided Pearson Chi-squared test. Continuous variables were compared using Student's t-test. Differences with a *p*-value <5% were considered significant.

## RESULTS

Five patients with endometriosis and infertility were included in the case group and four healthy fertile patients were enrolled in the control group. Table 1 shows patient demographic characteristics. In the case group, four patients had stage I endometriosis and one had stage II endometriosis.

**Table 1:** Demographic characteristics of women with infertility and endometriosis (case group) and healthy fertile women (control group).

	Control group (n=4)	Case group (n=5)	*p* value
Age (years)	35.2±2.2	30.4±4.3	.084^a^
Race			
Caucasian	3 (75%)	4 (80%)	0.86^b^
African-Brazilian	1 (25%)	1 (20%)	
BMI (Kg/m^2^)	26.6±2.8	22.8±4.1	.156^a^
Menarche (years)	11.5±1.3	12.6±1.1	.216^a^
AFC	12.5±1.0	13.6±5.9	.727^a^

All values are means±SD;

BMI = body mass index;

AFC = antral follicle count;

a Student’s t-test;

b Chi-squared test.

**Figure 1:**
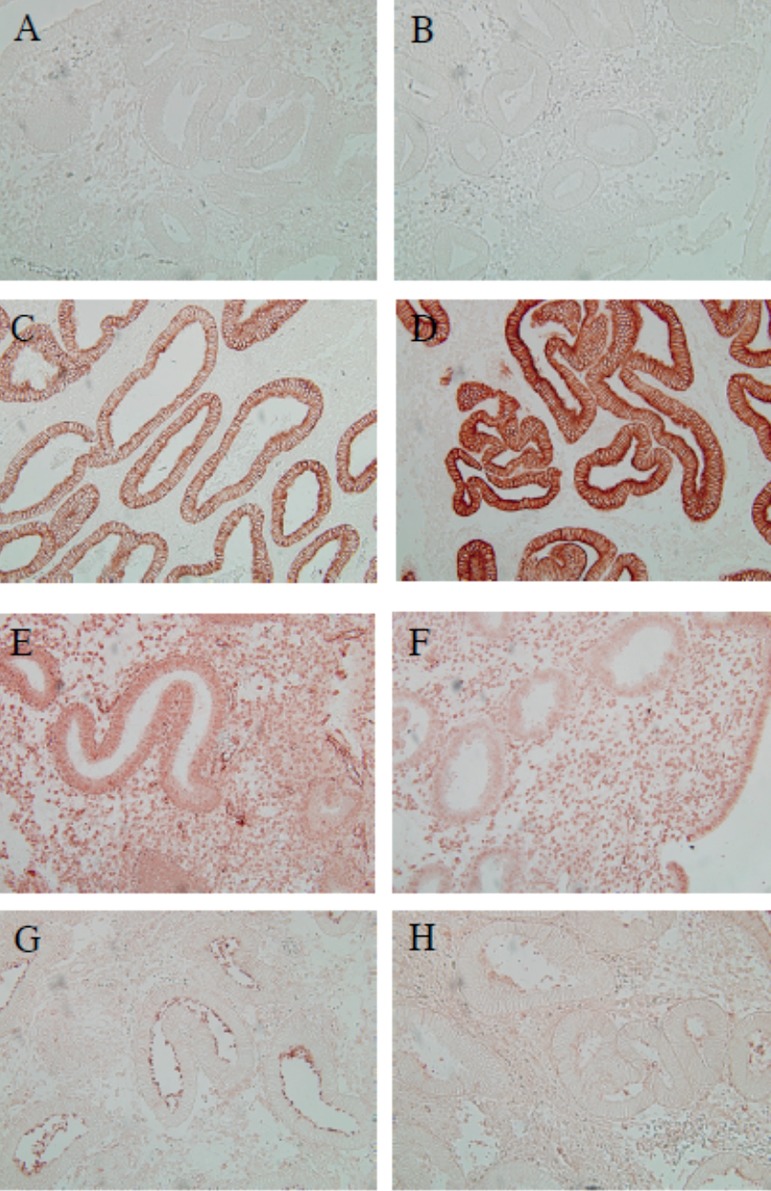
Immunohistochemical localization of MFG-E8, integrin αvβ3, and LIF in the human endometrium. Representative images of: (A) negative control from the control group (endometriosis and infertility), (B) and from the case group (healthy fertile patients); (C) MFG-E8 staining from the control group, (D) and from the case group; (E) Integrin αvβ3 staining from the control group, (F) and from the case group; and (G) LIF staining from the control group and from the case group (H).

Immunohistochemistry confirmed previous findings and revealed that MFG-E8 was predominantly located in glandular epithelium. MFG-E8´s receptor, integrin αvβ3, was localized in the epithelial, as well as in the stromal layer. Immunolocalization showed that LIF was preferentially observed in the luminal epithelium (Figure 1).

**Figure 2:**
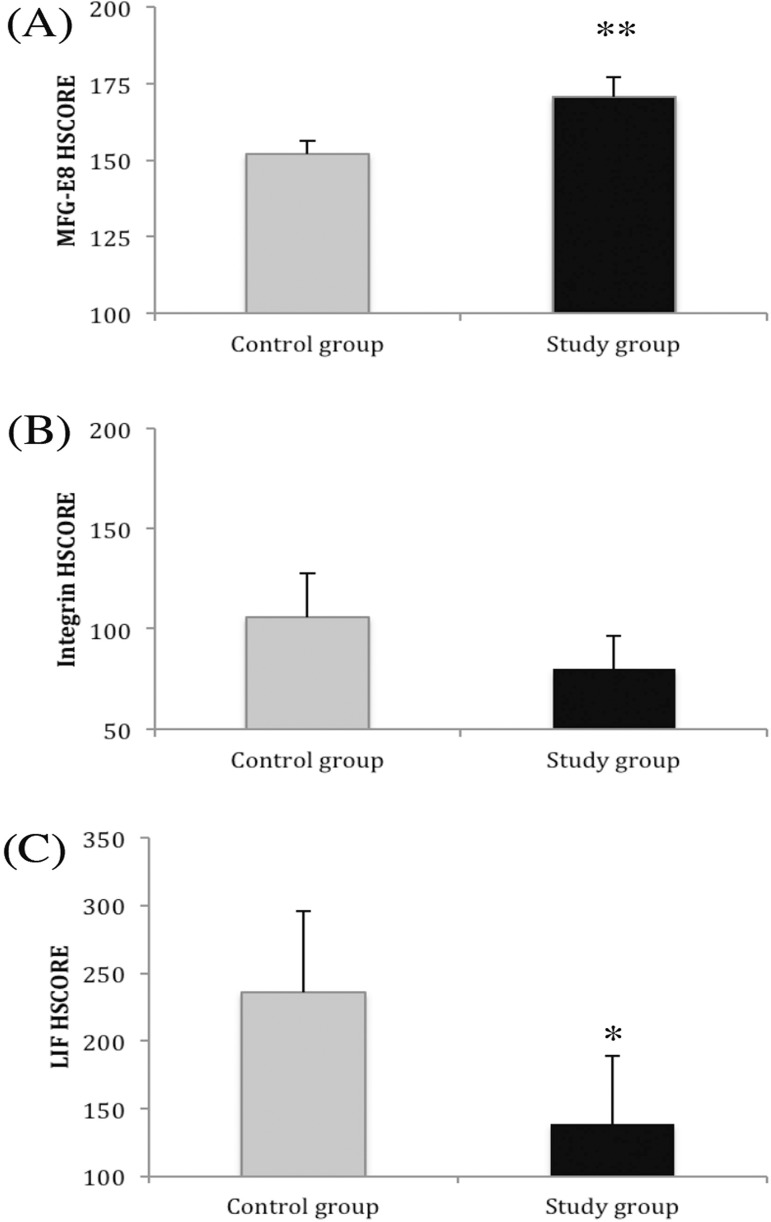
MFG-E8 (A), integrin αvβ3 (B) and LIF (C) HSCORE in healthy fertile patients (control group) and in patients with endometriosis and infertility. **p<0.001 compared with controls. *p<0.05 compared with controls.

Figure 2 shows the mean HSCORE for MFG-E8, integrin αvβ3 and LIF. Statistically significant differences were found for MFG-E8 (*p*<0.001) and LIF (*p*=0.033) between the control and case groups. No significant difference was found for integrin αvβ3 (*p*=0.084).

## DISCUSSION

This study showed for the first time that patients with endometriosis and infertility have increased MFG-E8 expression in the endometrium during the putative window of implantation. Patients also had decreased LIF expression during the same period. Nevertheless, no differences were found in the expression of integrin αvβ3 between the groups.

MFG-E8 is a glycoprotein identified for the first time in 1990 (Stubbs *et al*., 1990). Since then, it has been extensively studied in many physiological and pathological processes, especially in the immune system (Matsuda *et al*., 2011; Oba *et al*., 2011). A possible role for MFG-E8 in the implantation process was pointed out for the first time in 2005, when it was found up-regulated during the window of implantation (Mirkin *et al*., 2005). After that, other studies suggested its participation in the implantation process (Franchi *et al*., 2011; Schmitz *et al*., 2014); however, to the best of our knowledge, it had never been studied in the endometrium of infertile patients.

MFG-E8 is known to be involved in inflammatory processes (Komura *et al*., 2009), and it is regulated by TNF-α in the human endometrium (Yu *et al*., 2014). Moreover, endometriosis patients are known to present chronic inflammation (Zhao *et al*., 2015a; Berkes *et al*., 2014). Therefore, our hypothesis that endometriotic/infertile patients have increased MFG-E8 expression during the window of implantation was confirmed in the current study. We had previously shown in an in-vitro model that blocking MFG-E8 impairs the implantation process (Schmitz *et al*., 2014). Nevertheless, it seems that either the down regulation (Sinningen *et al*., 2015) or the up-regulation of MFG-E8 may impair physiological processes (Zhao et al., 2015b; Yamamoto *et al*., 2014).

MFG-E8 receptor integrin αvβ3 is a well-established endometrial receptivity marker. Nevertheless, its expression in the endometrium of individuals with endometriosis has been a topic of controversy in the literature. Our study found no differences in the expression of integrin αvβ3 between the groups, although levels tended to be lower in the case group (*p*=0.084). In 1994, a large study with 241 individuals with endometriosis showed that subjects with stage I/II endometriosis had decreased integrin expression (Lessey *et al*., 1994). However, the authors included biopsies after day 19 of the cycle, and not only during the window of implantation. Moreover, the study did not mention if all patients were infertile. On the other hand, two smaller studies failed to find such difference (Ordi *et al*., 2003; Casals *et al*., 2012) after analyzing integrin αvβ3 expression during the window of implantation.

A possible cause for the decrease in integrin αvβ3 expression is the fact that these patients had impaired HOXA10 production, which is responsible for the expression of subunit β3 (Taylor *et al*., 1999; Lu *et al*., 2013; Zhu *et al*., 2013). The overexpression of MFG-E8 may also down regulate the production of its receptor.

Another important endometrial receptivity marker that seems to be impaired in the endometrium of individuals with endometriosis is LIF. As also seen in our results, Dimitriadis *et al.* (2006) previously described diminished expression of LIF in patients with stage I/II endometriosis during the window of implantation. In addition, Alizadeh *et al.* (2011) also reported impaired LIF expression in a similar group of patients. Nevertheless, a study with 14 endometriosis patients showed no difference in LIF levels in uterine flushings when compared to fertile controls (21 patients) (Mikolajczyk *et al*., 2007).

The method used in this study is a modification of a previously published technique (Fuhrich *et al*., 2013) that allows for faster and more accurate results; the thresholding function in Metamorph^TM^ removes holes in background objects, thus allowing the quantification of thresholded areas only. Manually circumscribing objects as in Fuhrich et al., 2013 is much slower and may also include holes, which reduces the averaged staining intensity in a variable fashion depending on the individual object's hole area. Size filter settings in Metamorph^TM^ were also used to exclude stray pixels and small debris, enabling a more accurate measurement of cell structures. Unfortunately, not all types of staining can be adequately analyzed by this method, as integrin and LIF. This is why the traditional H-score was used in this study.

Although it may be argued that the study sample was relatively small, statistical significant differences were found. Moreover, the results agreed with what we were expecting, based on biological plausibility. Although we did not measure serum or urinary LH levels to further define the ovulation day, previous studies have defined ovulation only with ultrasound (Ecochard *et al*., 2013).

In conclusion, our study showed for the first time that patients with endometriosis and infertility have altered MFG-E8 expression in the endometrium during the putative window of implantation. Moreover, we also demonstrated that these patients have diminished LIF, as shown before. Nevertheless, there was no difference in the expression of integrin αvβ3, although in the case group levels tended to be lower. The endometrium of patients with endometriosis must be more thoroughly characterized to improve the understanding of the association between this condition and infertility.
